# Monoradiculopathy Caused by Sporadic Hemangioblastoma of the Conus Medullaris: Case Report and Literature Review

**DOI:** 10.7759/cureus.24099

**Published:** 2022-04-13

**Authors:** Ivo Kehayov, Polina Angelova, Ivan Batakliev, Veselin Belovezhdov, Borislav Kitov

**Affiliations:** 1 Department of Neurosurgery, Faculty of Medicine, Medical University of Plovdiv, Plovdiv, BGR; 2 Department of General and Clinical Pathology, Faculty of Medicine, Medical University of Plovdiv, Plovdiv, BGR; 3 Clinic of Neurosurgery, Sv. Georgi Hospital, Plovdiv, BGR

**Keywords:** surgery, intradural extramedullary, spinal, conus medullaris, hemangioblastoma

## Abstract

Sporadic spinal extramedullary hemangioblastomas of the conus medullaris are extremely rare. We present the case of a 40-year-old male with symptoms of severe back pain and monoradiculopathy. The magnetic resonance imaging (MRI) revealed an intradural extramedullary tumor attached to the conus medullaris. Total tumor removal was achieved via a typical posterior midline approach through laminectomy of L1 and L2 vertebrae, resulting in complete resolution of the preoperative symptoms. The histological examination was consistent with hemangioblastoma. To the best of our knowledge, this is the fifth case reported in the literature. We performed a brief literature review outlining the mainstay of diagnosis and therapeutic approach to these challenging lesions.

## Introduction

Hemangioblastomas represent 1-3% of all central nervous system tumors and are most frequently located in the cerebellum, brain stem, and spinal cord [1]. Most spinal hemangioblastomas are located in the cervical and thoracic areas but can also be found at the level of filum terminale and cauda equina, while conus medullaris localization is extremely rare [2-4].

Hemangioblastoma can be sporadic or associated with the von Hippel-Lindau syndrome with autosomal dominant inheritance caused by the VHL gene mutation in 3p25-26 chromosomes. The syndrome is also linked with other types of tumors such as retinal angiomatosis, endolymphatic sac neoplasms, kidney or pancreatic cancer, pheochromocytoma, etc. [5].

We present a case of sporadic hemangioblastoma in the area of the conus medullaris and cauda equina. We also conducted a brief literature review of the diagnostic and treatment approaches for this rare tumor location. To the best of our knowledge, this is the fifth reported case in the literature.

## Case presentation

**Figure 1:**
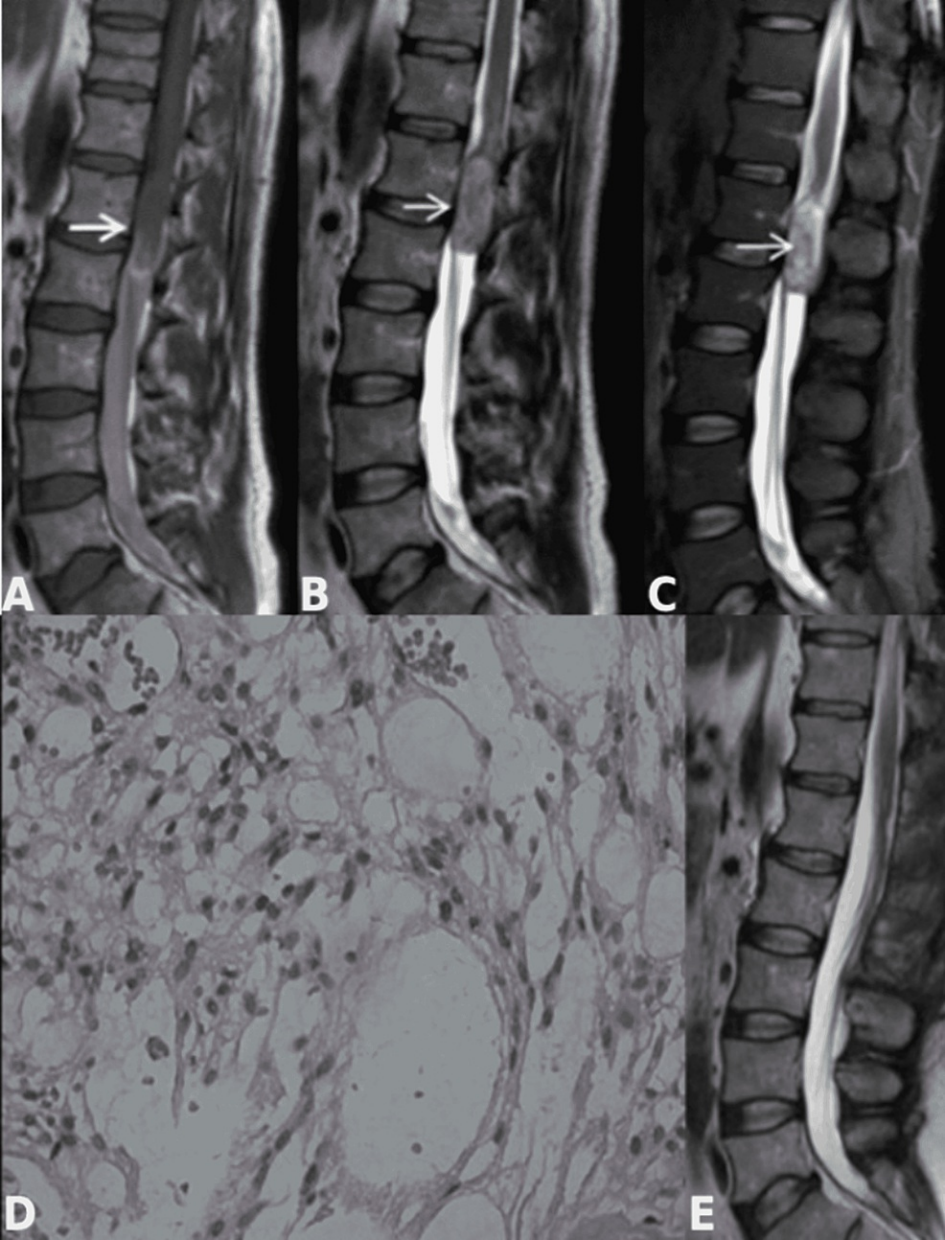
Imaging and histological diagnosis (A-C) Sagittal Т1-weighted, T2-weighted and turbo inversion recovery magnitude (TIRM) MRI images showing well-circumscribed intradural extramedullary neoplasm, measuring 4.5 cm × 1 cm, adherent to conus medullaris (arrows); (D) histological examination demonstrating tumor rich in stromal cells with small nuclei, large vacuoles, scattered red blood cells, thin-walled vessels and microhemorrhages (HE × 100); (E) postoperative sagittal T2-weighted MRI demonstrating total tumor removal.

We present the case of a 40-year-old male patient who presented with a gradual onset of progressive severe back pain for a duration of four months prior to hospital admission. The pain radiated towards the upper anterior surface of the right thigh. The symptoms were not relieved by conservative treatment with non-steroid anti-inflammatory drugs and were misinterpreted as a degenerative spinal disease. At hospital admission, the neurological examination revealed severe back pain, hyperalgesia, and hyperesthesia along the right L2 dermatome. The magnetic resonance imaging (MRI) revealed an intradural extramedullary tumor at the L1-L2 level, tightly adherent to the conus medullaris cranially and displacing the filum terminale and cauda equina roots to the right (Figure 1A-1C).

**Figure 2:**
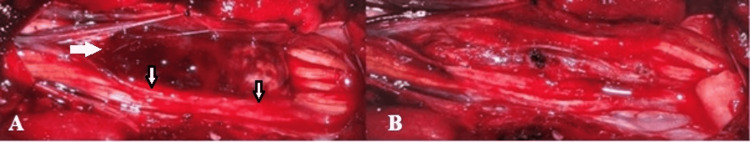
Intraoperative images (A) Tumor measuring 4.5 cm × 1 cm with fine capsule, originating from conus medullaris (white arrow) dislocating laterally the roots of cauda equina (black-and-white arrows); (B) magnified view after total tumor removal.

The patient was operated on via typical midline posterior surgical access through laminectomy of L1 and L2 vertebrae. The dura mater was found to be tense. After midline durotomy and arachnoid incision, we evacuated xanthochromatous cerebrospinal fluid. A cherry-red intradural extramedullary tumor with a subtle capsule was visualized that originated from the area of the conus medullaris and ventrally displaced the filum terminale. The surrounding roots of the cauda equina as well as the caudal and cranial poles of the tumor were identified (Figure 2A). Using a careful microsurgical technique in a step-by-step fashion, we first dissected the caudal pole of the tumor and relatively easily freed it from the surrounding nerve roots and filum terminale, which all remained intact. Only the cranial pole of the tumor necessitated meticulous separation due to its tight adherence to the conus medullaris. Finally, the tumor was totally removed en-bloc (Figure 2B).

The postoperative period was uneventful, with complete resolution of the preoperative symptoms. The histological examination revealed a tumor formation rich in stromal cells with small nuclei, large vacuoles, scattered red blood cells, and thin-walled vessels and microhemorrhages consistent with hemangioblastoma (Figure 1D). Given the histological findings, ophthalmoscopy, abdominal ultrasound, head and whole spine MRI were performed. No lesions typical of von Hippel-Lindau syndrome were found. The six-month follow-up confirmed normal neurological status and total tumor removal on the postoperative MRI (Figure 1E).

## Discussion

Sporadic intradural extramedullary hemangioblastomas of the conus medullaris are extremely rare, with only four cases reported in the literature worldwide (Table 1) [1,4,6,7].

**Table 1:** Overview of published cases with sporadic intradural extramedullary hemangioblastomas of the conus medullaris

Authors	Age	Gender	Clinical presentation	von Hippel-Lindau syndrome	Total resection of tumor	Complete recovery
Brisman et al. [6]	57	F	Lumbalgia and right leg radiculopathy	No	Yes	Yes
Dinc et al. [7]	33	F	Lumbalgia and right leg radiculopathy	No	Yes	Yes
Welling et al. [4]	43	М	Lumbalgia and legs dysesthesia	No	Yes	Yes
Shields et al. [1]	65	М	Lumbalgia and leg paresthesia	No	Yes	Yes
Our case	40	М	Lumbalgia and right leg radiculopathy	No	Yes	Yes

The age of the patients varied from 33 to 57 years, and our patient is no exception. Including the current case, the percentage of male patients prevails slightly, nearing 60%. Like others, our patient presented with pronounced lumbalgia, pain, and radiculopathy (Table 1). In all five cases, total tumor removal and complete postoperative recovery were achieved, which led to a definitive cure (Table 1).

Small-sized spinal cord hemangioblastomas in the area of the conus medullaris and cauda equina are usually asymptomatic. The neurological deficit occurs gradually with the increase in tumor size [1]. The most common symptoms are lumbalgia and radiculopathy, with hyperalgesia and dysesthesia radiating towards the legs. Myelopathy may occur with significant tumor growth associated with spinal cord and conus medullaris compromise [5]. Non-contrast and contrast-enhanced MRI are the gold standards for diagnosing hemangioblastomas [8]. In the T1 sequence, the tumor appears isointense, while in T2 it is moderately heterogeneous compared to the spinal cord. Generally, gadolinium application leads to intense enhancement of the tumor mass [8].

Sporadic intradural extramedullary hemangioblastomas are usually amenable to total surgical removal. In such cases, complete patient recovery is observed in over 96% of the cases [5]. Recurrence rates are higher in subtotal tumor resections or in tumors associated with von Hippel-Lindau syndrome [5].

The diagnosis of spinal hemangioblastomas is difficult due to overlapping symptoms with degenerative spinal diseases as well as the variety of neoplasms and vascular malformations (arteriovenous malformations, dural arteriovenous fistula, and cavernous malformation) that share similar MRI characteristics [1]. The neuroimaging differential diagnosis of an intradural extramedullary tumor in the area of conus medullaris and cauda equina includes a broad variety of spinal intradural lesions, such as nerve sheath tumors (schwannoma or neurofibroma), meningioma, paraganglioma, myxopapillary ependymoma, metastasis, and hemangioblastoma [9,10]. In contrast to intradural metastases, which are usually multiple, the rest of the neoplasms have similar neuroimaging findings and require further differentiation [8] (Table 2).

**Table 2:** Differential diagnosis of spinal hemangioblastomas and other spinal lesions based on their MRI characteristics

Differential diagnosis of spinal hemangioblastoma	T1 MRI	T2 MRI	T1 C+ (Gd) MRI
Spinal hemangioblastoma	Hypo- to isointense	Iso- to hyperintense; surrounding edema; associated syrinx	Vivid enhancement
Spinal meningioma	Isointense to slightly hypointense	Isointense to slightly hyperintense	Moderate homogeneous enhancement
Mixopapillary epedymoma	Isointense	Overall high intensity	Enhancement is virtually always seen
Leptomeningeal spinal metastases	Isointense thickened nerve roots or nodular lesions	Spinal cord edema	Enhancing tumor nodules on the spinal cord, nerve roots or cauda equina;
Spinal schwannoma	75% are isointense, 25% are hypointense	Hyperintense, often with mixed signal	Enhancement is virtually always seen
Spinal neurofibroma	Hypointense	Hyperintense	Heterogenous enhancement
Spinal paraganglioma	Isointense	Hyperintense	Enhancement is virtually always seen
Spinal cord metastases	Hypointense	Hyperintense	Avid enhancement in >80% of cases
Spinal astrocytoma	Isointense to hypointense	Hyperintense	Vast majority enhance; patchy enhancement pattern
Spinal ependymoma	Isointense to hypointense	Hyperintense	Enhance strongly, somewhat inhomogeneously
Spinal arteriovenous malformations	Signal voids from high-velocity flow; dilated perimedullary vessels	Signal voids from high-velocity flow; increased cord signal	N/A
Spinal dural arteriovenous fistula	Intramedullary hypointensity and flow voids on the cord surface	Diffuse multilevel intramedullary hyperintensity (edema); hypointensity in the periphery of the cord; prominent serpiginous intradural extramedullary flow voids - most specific finding	Patchy intramedullary enhancement; serpentine enhancing veins on the cord surface
Spinal cavernous malformation	Rounded regions of heterogeneous signal intensity	Rounded regions of heterogeneous signal intensity; low signal intensity rim	Minimal enhancement

The preoperative differentiation of spinal tumors from vascular abnormalities is of particular importance. In such cases, selective spinal angiography is a useful tool for the diagnosis of suspected vascular malformations. It can also be used to perform preoperative embolization, which reduces intraoperative bleeding [7,11].

In our case, we present the difficulties in the differential diagnosis of sporadic intradural extramedullary hemangioblastoma of the conus medullaris. In the same way as the case described by Shields et al., the suggested preoperative MRI diagnosis was ependymoma or schwannoma [1], while the final histological examination revealed hemangioblastoma.

## Conclusions

The differential diagnosis of tumors in the area of conus medullaris and/or cauda equina should include hemangioblastomas. Selective spinal angiography could facilitate the differentiation between spinal tumors and vascular pathology. In cases with proven spinal hemangioblastomas, a screening for von Hippel-Lindau syndrome should be performed. MRI is the imaging method of choice for accurate preoperative topical diagnosis and adequate surgical planning. The aim of the surgery must be total tumor removal, which provides permanent neurological recovery for these patients and reduces recurrence rates.
